# Interpreting Resting Energy Expenditure in Critically Ill Patients with Obesity: Clinical Impact of Weight Adjustment

**DOI:** 10.3390/jcm15051677

**Published:** 2026-02-24

**Authors:** Sebastián Chapela, Jaen Cagua-Ordoñez, Juan Marcos Parise-Vasco, Daniel Tettamanti Miranda, Claudia Kecskes, Natalia Llobera, Jesica Asparch, Mariana Rella, María Victoria Peroni, Martha Montalvan, María Jimena Reberendo, Facundo Gutierrez, Mario O. Pozo, Ludwig Álvarez-Córdova, Daniel Simancas-Racines

**Affiliations:** 1 Departamento de Bioquímica, Facultad de Medicina, Universidad de Buenos Aires, Buenos Aires C1121ABG, Argentina; sebachapela@gmail.com; 2 Unidad de Soporte Nutricional, Hospital Británico de Buenos Aires, Buenos Aires C1280AEB, Argentina; naty.llobera@gmail.com (N.L.); jimereberendo@gmail.com (M.J.R.); 3 Escuela de Medicina, Pontificia Universidad Católica del Ecuador, Santo Domingo 230203, Ecuador; jccagua@pucesd.edu.ec; 4 Facultad de Ciencias de la Salud y Bienestar Humano, Universidad Tecnológica Indoamérica, Quito 170103, Ecuador; jmparise@puce.edu.ec; 5 Facultad de Salud y Bienestar, Pontificia Universidad Católica del Ecuador, Quito 170143, Ecuador; dasimancas@puce.edu.ec; 6 Carrera de Medicina, Universidad Católica de Santiago de Guayaquil (UCSG), Guayaquil 090615, Ecuador; dantettamanti@gmail.com; 7 Sección de Soporte Nutricional, Hospital Italiano, Buenos Aires C1439BSN, Argentina; claudia.kecskes@hospitalitaliano.org.ar (C.K.); jesica.asparch@gmail.com (J.A.); 8 Universidad del Hospital Italiano, Buenos Aires C1199ABD, Argentina; 9 Servicio de Endocrinología, Nutrición y Diabetes, Hospital Británico de Buenos Aires, Buenos Aires C1280AEB, Argentina; marianarella2024@gmail.com; 10 Hospital Italiano de Buenos Aires, Buenos Aires C1199ABD, Argentina; mv.peroni@hotmail.com; 11 Escuela de Medicina, Universidad Espíritu Santo, Samborondón 0901952, Ecuador; mmontalvanmd53@gmail.com; 12 Servicio de Terapia Intensiva, Hospital Británico de Buenos Aires, Buenos Aires C1280AEVB, Argentina; facundo_gutierrez@yahoo.com.ar (F.G.); pozomario@hotmail.com (M.O.P.); 13 Maestría de Nutrición y Dietética, Facultad de Ciencias de la Salud, Universidad de Las Américas (UDLA), Quito 170124, Ecuador; 14 Facultad de Ciencias de la Salud y Bienestar Humano, Universidad Tecnológica Indoamerica, Ambato 180150, Ecuador

**Keywords:** indirect calorimetry, obesity, critical care, respiratory quotient, resting energy expenditure

## Abstract

**Background:** Accurately estimating resting energy expenditure (REE) in critically ill obese patients remains a significant clinical challenge, as predictive equations are consistently inadequate. Metabolic heterogeneity across obesity classes and the role of substrate utilization are insufficiently characterized. **Objective:** To evaluate the impact of different weight-normalization methods on the interpretation of REE and to identify independent metabolic determinants of weight-adjusted energy expenditure in critically ill patients with obesity. **Methods:** Bicentric cross-sectional study of 148 critically ill adults with obesity undergoing indirect calorimetry. REE normalized by actual body weight (REE/kg), ideal body weight (REE/IBW), and adjusted body weight (REE/AdjBW) was calculated. Multivariable models with robust standard errors (HC3), stratified analyses by obesity class (I–III) with a Chow test, and internal validation were performed using 10-fold cross-validation and bootstrap resampling (1000 iterations). **Results:** Absolute REE did not differ significantly between BMI categories (*p* = 0.679), while REE/kg progressively decreased from normal weight (27.8 kcal/kg/day) to class III obesity (16.9 kcal/kg/day; *p* < 0.001). The respiratory quotient (RQ) emerged as the most robust independent correlate of adjusted REE (β = −13 to −15 kcal·kg^−1^·day^−1^; *p* < 0.001), whereas clinical severity scores (SOFA, APACHE II) and comorbidity (Charlson) did not show significant associations. Stratified analyses revealed significant structural heterogeneity between obesity classes (F = 4.545, *p* = 0.0001), with no significant predictors identified in class III obesity, likely reflecting limited statistical power in this subgroup. **Conclusions:** Normalizing REE using different weight indices fundamentally alters its metabolic interpretation. RQ surpasses traditional clinical scores as a correlate of adjusted REE, consistent with a phenotype of metabolic inflexibility. The heterogeneity between obesity classes underscores the need for individualized indirect calorimetry rather than reliance on predictive equations.

## 1. Introduction

Obesity is an escalating global public health problem, with rates having tripled since 1975 and currently affecting more than 650 million adults worldwide [1]. In the intensive care unit (ICU), the prevalence of obesity is high, reported in 20% to 36% of critically ill patients [2,3]. However, determining their energy requirements remains an unresolved challenge in critical care. Critically ill patients with obesity exhibit paradoxical metabolic responses: those with moderate obesity show survival advantages despite increased complications, including acute kidney injury, whereas individuals with severe obesity (BMI ≥ 40 kg/m^2^) face an elevated risk of mortality [1,2,4].

Chronic nutrient excess in obesity induces insulin resistance and altered signaling, driven by acyl-CoA accumulation, serine phosphorylation, and reduced activation of protein kinase B (Akt/PKB) [5]. The intersection of chronic nutrient overload and acute stress manifests as metabolic inflexibility, the inability of the organism to efficiently switch between carbohydrate and lipid oxidation in response to substrate availability [5,6]. Mitochondria are central to achieving such flexibility. In the context of obesity, metabolic inflexibility is characterized by mitochondrial overload and incomplete fatty acid oxidation, a phenomenon known as “metabolic gridlock” due to elevated acetyl-CoA levels [7]. The persistent oxidation of mixed carbon fuels in people with metabolic inflexibility increases the risk of metabolic injury. Consequently, the critically ill patient with obesity presents volatile metabolic profiles, ranging from acute hypometabolism to hypermetabolism, further aggravated by underlying chronic inflexibility, rendering accurate estimation of resting energy expenditure (REE) challenging for optimizing nutritional therapy [5,8].

During the first 48 h of critical illness, patients experience a significant increase in REE that develops early and persists progressively even after hospital discharge, reflecting the intensity of underlying physiological stress [8,9,10]. This pattern of sustained hypermetabolism is even more pronounced in patients with obesity, who exhibit substantial elevations in their energy requirements that predictive equations fail to capture consistently [11]. The clinical consequences of this mismeasurement are severe: underfeeding increases the risk of nosocomial infections and prolongs mechanical ventilation, whereas overfeeding leads to hyperglycemia, hepatic steatosis, and difficulty with ventilator weaning [12]. Obese patients already experience doubled infection rates and may require an additional 1.5 to 3 days of mechanical ventilation, and remain 1 to 3.5 days longer in the ICU than normal-weight patients, representing a substantial use of resources that could potentially be optimized with accurate metabolic assessment [13].

The application of predictive equations such as the Harris–Benedict, Penn State, and Mifflin–St Jeor equations in obese patients is complicated by the debate over which body weight variable should be used in the calculations [14,15]. Indirect calorimetry is the reference method for this assessment, yet its routine use in clinical practice faces significant constraints related to cost, logistics, and the need for specialized personnel [16]. For this reason, healthcare professionals commonly use predictive equations to estimate energy requirements. However, the accuracy of these equations, whether developed for healthy populations or critically ill patients, has been shown to decrease progressively with increasing obesity severity [17]. Available evidence indicates that these equations tend to systematically underestimate true caloric needs, with alarmingly low precision in mechanically ventilated critically ill patients [17,18]. The application of these formulas in obese patients is further complicated by ongoing controversy regarding which body weight variable (actual, ideal, or adjusted) should be used, with no robust evidence supporting one method over another. This challenge is exacerbated by the scarcity of studies using indirect calorimetry to validate equations in patients with severe or morbid obesity. This minority population requires highly specialized therapeutic approaches [19].

The inherent complexity of metabolic assessment in the intensive care unit raises additional questions regarding the interpretation of parameters derived from indirect calorimetry [15,19]. The respiratory quotient (RQ), an essential component of calorimetric measurements that reflects substrate utilization and determines the physiological validity of the readings, requires particularly cautious interpretation in the context of the critically ill obese patient [17]. The extreme metabolic variability characteristic of this population, exacerbated by the metabolic inflexibility typical of obesity and multiple pharmacological interventions, highlights the urgent need for rigorous methodological consensus to ensure the validity of calorimetric measurements [15,18].

In the absence of a consensus in clinical guidelines on whether to use actual, ideal or adjusted body weight when calculating nutritional requirements, it is crucial to consider the clinical implications of applying body weight adjustments when interpreting indirect calorimetry results for the individualized nutritional management of critically ill obese patients [16]. In light of this issue, the objective of this study is to evaluate the clinical implications of different body weight adjustment methods for interpreting REE in critically ill patients with obesity.

## 2. Methods

### 2.1. Study Design and Population

This was a bicentric, retrospective, analytical cross-sectional study conducted in two tertiary-level intensive care units (ICUs) providing high-complexity patient care. The study included all consecutive adult patients with critical illness who underwent indirect calorimetry (IC) as part of routine clinical metabolic assessment between January 2016 and January 2021. Indirect calorimetry was integrated into standard nutritional evaluation practices in both ICUs and was not performed for research purposes. Each record represented a unique patient; therefore, repeated measurements were not included. The present analyses form part of a series of articles derived from the same retrospectively approved bicentric research framework, each addressing distinct research questions using complementary analytical approaches [20].

Adult patients (≥18 years) admitted to the ICU during the study period were eligible for inclusion if indirect calorimetry was performed during critical illness. Only the first valid indirect calorimetry measurement per patient was included to ensure independence of observations. No a priori sample size calculation was performed due to the retrospective design of the study; all eligible patients meeting inclusion criteria during the study period were included. This approach corresponds to a census of all eligible patients undergoing indirect calorimetry during the study period.

Patients were excluded if indirect calorimetry data were incomplete or technically invalid, or if measurements were obtained under conditions known to substantially compromise accuracy (e.g., significant air leaks or extracorporeal support). One underweight patient was excluded because it constituted a single extreme value that precluded meaningful group-based comparisons. A total of 148 eligible patients were included in the final analysis.

Patients were categorized according to the World Health Organization (WHO) BMI classification as normal weight (18.5–24.9 kg/m^2^), overweight (25.0–29.9 kg/m^2^), obesity grade I (30.0–34.9 kg/m^2^), obesity grade II (35.0–39.9 kg/m^2^), or obesity grade III (≥40.0 kg/m^2^) [21].

### 2.2. Data Collection and Variables

Clinical, demographic, and metabolic data were extracted from electronic medical records and the corresponding calorimetry device logs. The variables collected included:

Demographic and clinical data: age (years), sex, BMI (kg/m^2^), Sequential Organ Failure Assessment (SOFA) score, Acute Physiology and Chronic Health Evaluation II (APACHE II) score, and Charlson Comorbidity Index (CCI).

Calorimetric parameters: resting energy expenditure (REE, kcal/day), oxygen consumption (VO_2_, mL/min), carbon dioxide production (VCO_2_, mL/min), and respiratory quotient (RQ = VCO_2_/VO_2_).

The derivative indices were computed to express REE normalized by different weight metrics:

- REE/kg: REE per kilogram of actual body weight (kcal/kg/day);
- REE/IBW: REE per kilogram of ideal body weight (IBW), calculated using the Miller formula;
- REE/AdjBW: REE per kilogram of adjusted body weight, defined as IBW + 0.25 × (actual—IBW).

All calorimetric measurements were made under thermoneutral conditions during mechanical ventilation. Hemodynamic and respiratory stability were confirmed prior to measurement. Each test lasted 20–30 min and steady-state periods (<10% variability in VO_2_ and VCO_2_, and <5% in RQ) were averaged. Data were validated by requiring RQ values between 0.67 and 1.3 to ensure physiological plausibility.

### 2.3. Statistical Analysis

#### 2.3.1. Descriptive and Comparative Analyses

All analyses were conducted using R (version 4.5). Continuous variables were summarized as mean ± standard deviation (SD) when normally distributed, or as median and interquartile range (IQR, Q1–Q3) when distributions were non-normal, as assessed using the Shapiro–Wilk test. Categorical variables were expressed as counts and percentages.

For normally distributed continuous variables, group comparisons across BMI categories would have been performed using one-way analysis of variance (ANOVA). However, normality testing demonstrated non-normal distributions for all REE-related variables (REE *p* = 0.0013; REE/kg *p* < 0.0001; REE/IBW *p* = 0.0009; REE/ADJBW *p* = 0.0044); therefore, non-parametric inferential tests were applied. Comparisons across BMI categories were conducted using the Kruskal–Wallis test, followed by Dunn–Bonferroni post hoc corrections for multiple pairwise comparisons when the overall test was statistically significant.

The correlations between the calorimetric and clinical parameters were examined using the Spearman rank correlation coefficient (ρ), with the strength of the correlation interpreted as weak (|ρ| < 0.3), moderate (0.3 ≤ |ρ| < 0.7), or strong (|ρ| ≥ 0.7). All tests were two-tailed, and statistical significance was established at *p* < 0.05.

Statistical analyses and tables were generated using R (version 4.5.0; R Foundation for Statistical Computing, Vienna, Austria) through reproducible workflows implemented in the gtsummary, rstatix, and ggplot2 packages, ensuring standardized and transparent reporting.

#### 2.3.2. Multivariable Analysis

- Modeling Strategy and Outcomes

A series of multivariable models were developed to identify the independent determinants of REE and its normalized forms. The primary dependent variable was REE normalized by ideal body weight (REE/IBW, kcal·kg^−1^·day^−1^). Secondary outcomes included REE/kg (per actual weight) and REE/AdjBW (per adjusted weight), to evaluate whether weight correction modifies the interpretation of energy metabolism in obesity phenotypes. All models were estimated using ordinary least squares (OLS) with Huber–White heteroskedasticity-consistent (HC3) standard errors to correct for variance non-homogeneity.

The general form of the primary model was:

$${\left(REE/IBW\right)}_{i}=\beta_{0}+\beta_{1}{\left(BMI\right)}_{i}+\beta_{2}{\left(RQ\right)}_{i}+\beta_{3}{\left(VO_{2}\right)}_{i}+\beta_{4}{\left(VCO_{2}\right)}_{i}+\beta_{5}{\left(Age\right)}_{i}+\beta_{6}{\left(Sex\right)}_{i}\;+\beta_{7}{\left(SOFA\right)}_{i}+\beta_{8}{\left(APACHEII\right)}_{i}+\beta_{9}{\left(Charlson\right)}_{i}+\varepsilon_{i}$$

where $\varepsilon_{i}$ denotes the random error term with zero mean and heteroskedastic variance.

Linearity was verified by component–residual plots, and normality of residuals by Q–Q plots. Collinearity was evaluated using variance inflation factors (VIF < 5), and alternative specifications excluding collinear predictors were tested to ensure the stability of the model.

- Stratified Regression by Obesity Category

To explore whether REE determinants differed between obesity grades, stratified models were fitted independently for obesity grades I, II, and III. This approach allowed estimation of the heterogeneity of the coefficients associated with VO_2_, VCO_2_, and RQ, thereby testing whether the metabolic drivers of energy expenditure vary with adiposity severity.

Structural differences between strata were formally tested using the Chow test:

$$F=\frac{\left(S_{p}-\left(S_{1}+S_{2}+S_{3}\right)\right)/k}{\left(S_{1}+S_{2}+S_{3}\right)/\left(n_{1}+n_{2}+n_{3}-3k\right)}$$

where $S_{p}$ is the residual sum of squares from the pooled model, $S_{j}$ represents the sum of squared residuals in each subgroup, $n_{j}$ the sample size per subgroup, and k the number of estimated parameters. A significant *p* value (*p* < 0.05) indicated structural non-equivalence among obesity strata.

- Interaction Models and the Hypothesis of Metabolic Inflexibility

To evaluate whether the effect of adiposity on energy metabolism was modified by substrate oxidation or comorbidity burden, pre-specified interactions were incorporated: BMI × RQ and BMI × Charlson. The expanded model took the form:

$${\left(REE/IBW\right)}_{i}=\beta_{0}+\beta_{1}{\left(BMI\right)}_{i}+\beta_{2}{\left(RQ\right)}_{i}+\beta_{3}{\left(Charlson\right)}_{i}+\beta_{4}{\left(BMI\times RQ\right)}_{i}\;+\beta_{5}{\left(BMI\times Charlson\right)}_{i}+Covariables+\varepsilon_{i}$$

Significant interactions were explored through adjusted marginal effects and partial prediction graphs generated using R (version 4.5.0; R Foundation for Statistical Computing, Vienna, Austria) with the ggeffects and emmeans packages, allowing a graphical interpretation of how the BMI–REE association changes according to RQ or comorbidity level.

- Nonlinear Models with Restricted Cubic Splines

Given prior evidence of nonlinearity between adiposity and energy expenditure, restricted cubic splines (RCS) were used to model BMI flexibly. Splines were fitted with three degrees of freedom (df = 3) and knots at the 10th, 50th, and 90th percentiles of BMI distribution:

$${\left(REE/IBW\right)}_{i}=\beta_{0}+f_{RCS}{\left(BMI\right)}_{i}+Covariables+\varepsilon_{i}$$

where $f_{RCS}(BMI)$ denotes the smooth non-linear transformation of BMI. This parameterization permitted detection of inflection points at which additional increases in BMI ceased to be associated with higher adjusted energy expenditure.

#### 2.3.3. Sensitivity Analyses

Model robustness was examined through multiple predefined sensitivity analyses:

1. Exclusion of observations with RQ outside the physiological range (0.75–1.05).
2. Reestimation of models after removing extreme BMI values (>60 kg/m^2^).
3. Evaluation of the incremental predictive value of the Charlson index, by comparing models with and without its inclusion (ΔAICc > 2 indicating substantial improvement).
4. Reclassification of the Charlson index into three categorical levels (low, moderate, high) to test the consistency of its effect.
5. Estimation of alternative bioenergetic efficiency models using REE/VO_2_ and REE/VCO_2_ as dependent variables.
6. Comparison of complete-case models versus multiple imputation by chained equations (MICE, m = 20).

#### 2.3.4. Model Validation and Performance Assessment

All final models were internally validated using 10-fold cross-validation (k = 10) and bootstrap resampling (1000 iterations) to evaluate coefficient stability and minimize overfitting. Predictive accuracy was quantified through complementary indicators: the adjusted R^2^ (explained variance), root mean square error (RMSE), mean absolute error (MAE), and mean absolute percentage error (MAPE), each reflecting different aspects of model precision and residual variability.

Model calibration was examined by regressing predicted against observed REE values, obtaining the slope and intercept as measures of systematic bias and proportional accuracy. Ideal calibration corresponded to a slope of 1 and an intercept of 0, indicating perfect agreement. Additionally, decile-based calibration plots were used to visually assess prediction accuracy across the entire outcome distribution, ensuring consistent performance throughout the prediction range.

### 2.4. Software and Reproducibility

All analyses were performed in R (version 4.5.0; R Foundation for Statistical Computing, Vienna, Austria) using the following packages with their respective versions (CRAN): gtsummary 2.5.0, mice 3.19.0, emmeans 2.0.1, and other commonly used packages (stats, sandwich, lmtest, splines, quantreg, car, performance, ggeffects, caret, rsample, yardstick, glmnet, gt).

Although data were collected from two centers, identifiers were unavailable; thus, clustering by site could not be modeled. However, robust (HC3) variance estimators were applied to ensure valid inference in the presence of heteroskedasticity or inter-center variability. All scripts were version-controlled, ensuring full reproducibility.

## 3. Results

### 3.1. Patient Characteristics and Energy Expenditure Profile

**Figure 1:**
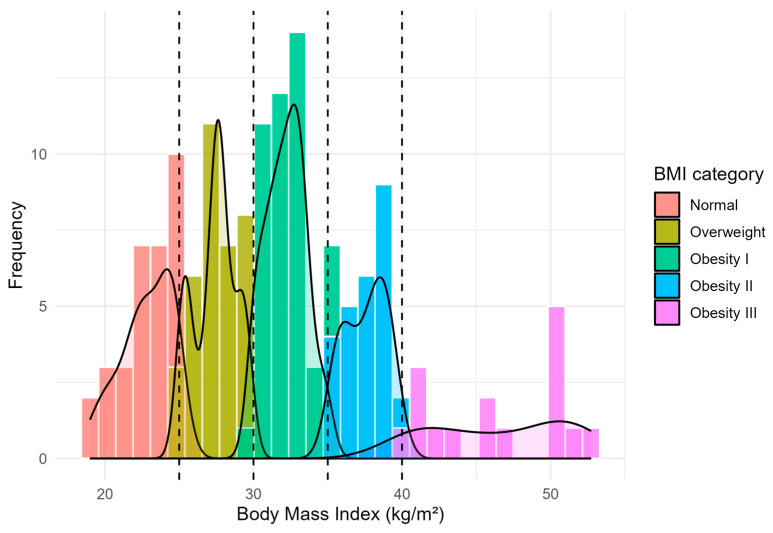
Distribution of BMI and obesity categories among critically ill patients. Histogram and kernel density curve showing BMI distribution across the study population (*n* = 148). Vertical dashed lines delineate standard BMI cut-offs for overweight (25 kg/m^2^) and obesity classes I (30 kg/m^2^), II (35 kg/m^2^), and III (40 kg/m^2^). Color-coded bars correspond to the following categorical classifications: normal weight, overweight, and obesity I–III.

A total of 148 critically ill patients were included after excluding one underweight outlier. The median age was 66 years (IQR: 54–74), and 56% were male. According to BMI, 19.6% were normal weight, 23.0% overweight, 29.7% obesity class I, 16.9% obesity class II, and 10.8% obesity class III (Figure 1).

Mean REE was 1874 ± 507 kcal/day and did not differ significantly across BMI categories. In contrast, REE normalized per kilogram of actual body weight (REE/kg) decreased progressively with increasing BMI (overall median 20.5 [17.1–25.7] kcal/kg/day; *p* < 0.001), whereas REE normalized by ideal (REE/IBW) or adjusted body weight (REE/AdjBW) showed no significant intergroup differences.

Mean VO_2_ was 265.9 ± 76.5 mL/min and median VCO_2_ was 218.8 (189.0–294.6) mL/min. The respiratory quotient (RQ) averaged 0.9 ± 0.2 and differed significantly across BMI categories (*p* < 0.001). Clinical severity scores (SOFA, APACHE II) and comorbidity burden (Charlson index) were broadly comparable between groups, although patients with obesity class III had higher SOFA scores.

### 3.2. Differences in Energy Expenditure and Metabolic Patterns Across BMI Categories

The comparative analysis (Table 1) identified significant differences in REE/kg (*p* < 0.001), RQ (*p* < 0.001), SOFA (*p* = 0.002), and BMI (*p* < 0.001), whereas REE (*p* = 0.679), REE/IBW (*p* = 0.139), REE/ADJBW (*p* = 0.712), VO_2_ (*p* = 0.405), VCO_2_ (*p* = 0.298), APACHE II (*p* = 0.517), age (*p* = 0.540), and Charlson index (*p* = 0.192) showed no significant differences between groups.

**Figure 2:**
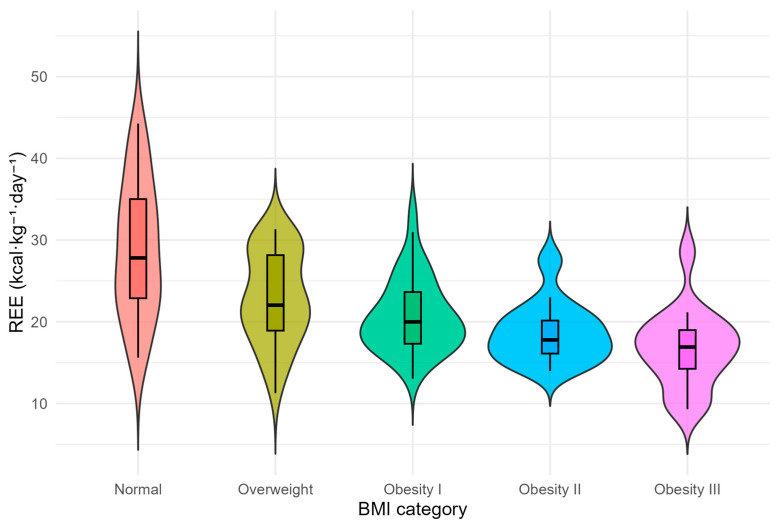
Resting energy expenditure normalized per actual body weight (REE/kg) across BMI categories. Violin and box plots showing the distribution of REE (kcal·kg^−1^·day^−1^) across BMI-defined groups. Median REE/kg decreased progressively with increasing adiposity (Kruskal–Wallis *p* < 0.001), indicating reduced relative metabolic activity in higher BMI strata.

Post hoc Dunn–Bonferroni comparisons indicated that REE/kg was significantly higher in the normal-weight group than in obesity grades I–III (all Bonferroni-adjusted *p* < 0.001). In addition, RQ was significantly lower in obesity grades I–III compared with the overweight group (Bonferroni-adjusted *p* = 0.001–0.020), and it was also lower in obesity grade I compared with the normal-weight group (Bonferroni-adjusted *p* = 0.017), suggesting a shift toward relatively lower RQ values with increasing adiposity (Figure 2).

Additionally, patients with class III obesity showed significantly higher SOFA scores than those with class I or II obesity (*p* = 0.005 and *p* = 0.006, respectively), indicating greater organ dysfunction severity among the most obese patients. No significant differences were observed in APACHE II, age, or Charlson index across BMI categories.

### 3.3. Associations Between Energy Expenditure, Body Composition, and Clinical Status

Spearman correlation analysis demonstrated strong interrelationships between energy expenditure indices and respiratory parameters. There were strong positive correlations between REE_PI and REE_PA (r = 0.95), REE_PI and VO_2_ (r = 0.87), and REE_PA and VCO_2_ (r = 0.81), confirming the internal consistency of indirect calorimetry-derived measurements.

Weight-normalized energy expenditure exhibited a distinct pattern. REE/kg correlated moderately with VCO_2_ (r = 0.67) and inversely with BMI (r = −0.56), indicating that increasing adiposity was associated with lower relative energy expenditure per kilogram of actual body weight. Age and the Charlson comorbidity index showed mild negative correlations with energy expenditure measures (r ranging from −0.26 to −0.34), whereas illness severity scores were weakly related to metabolic indices. SOFA and APACHE II were moderately correlated with each other (r = 0.55), but their associations with calorimetric variables were limited.

Overall, these findings indicate that absolute energy expenditure is primarily anchored to gas exchange physiology, whereas the apparent variation in metabolic rate emerges mainly after weight normalization and tracks with adiposity and, to a lesser extent, chronic comorbidity and age (Table 1).

### 3.4. Determinants of Resting Energy Expenditure and the Impact of Weight Adjustment Across Obesity Phenotypes

In multivariable OLS models with heteroskedasticity-consistent (HC3) standard errors, the respiratory quotient (RQ), male sex, and carbon dioxide production (VCO_2_) consistently emerged as independent determinants of weight-normalized REE across all normalization approaches (REE/kg, REE/IBW, and REE/AdjBW). Higher RQ values were strongly associated with lower normalized REE (β ≈ −13 to −15 kcal·kg^−1^·day^−1^; *p* < 0.001), while VCO_2_ was a robust positive predictor (β ≈ 0.07–0.09; *p* < 0.001), consistent with its direct contribution to energy expenditure estimation. VO_2_ showed a smaller but statistically significant positive association in the REE/IBW and REE/AdjBW models (β ≈ 0.016–0.020; *p* < 0.05).

Male sex was independently associated with lower normalized REE (β ≈ −3.7 to −4.7 kcal·kg^−1^·day^−1^; *p* < 0.001). This effect pertains to weight-normalized outcomes and should not be interpreted as a lower absolute REE. In contrast, BMI, age, illness severity (SOFA, APACHE II), and comorbidity burden (Charlson index) were not independently associated with adjusted REE in the main models, suggesting that metabolic and gas exchange variables explain most interindividual variability.

**Figure 3:**
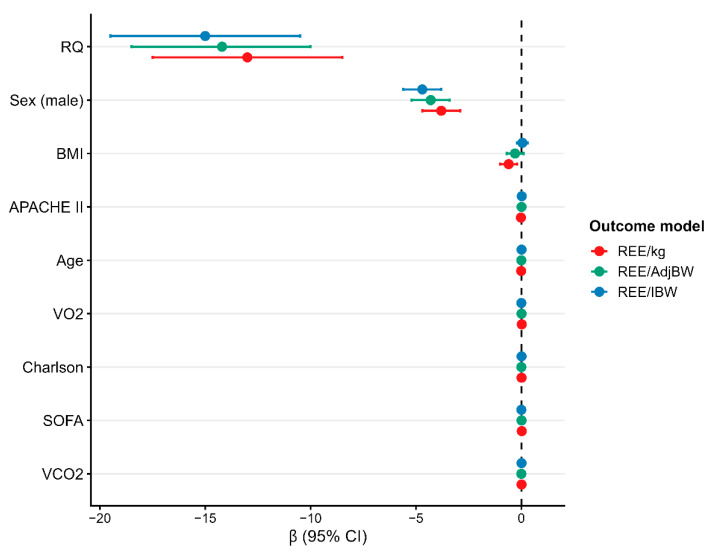
Comparative adjusted effects across multivariable models for resting energy expenditure. Forest plot showing adjusted β coefficients (95% CI) for key determinants across three models (REE/kg, REE/ADJBW, REE/IBW). The respiratory quotient (RQ) and male sex consistently exhibit negative associations with adjusted REE, whereas VCO_2_ remains a robust positive predictor across all specifications.

Collinearity assessment showed low-to-moderate VIF values for most predictors (VIF < 3.5). As expected, VO_2_ and VCO_2_ showed higher VIFs due to their physiological interdependence and their role in the Weir equation and RQ computation. Importantly, alternative specifications excluding VO_2_ or VCO_2_ did not materially change the magnitude or significance of the main predictors (RQ and sex), and overall model fit remained stable (R^2^ = 0.86; ΔAICc < 2). Model diagnostics supported adequate linearity and residual behavior (Figure 3).

### 3.5. Metabolic Determinants of Energy Expenditure Across Obesity Grades

Stratified multivariable analyses suggested heterogeneous metabolic profiles across obesity grades. In obesity class I, RQ remained a strong negative determinant of adjusted REE/IBW (β = −15.87; *p* = 0.001), while VCO_2_ showed the most robust positive association (β = 0.11; *p* < 0.001). Male sex was also independently associated with lower adjusted REE (β = −3.61; *p* < 0.001), whereas SOFA, APACHE II, and Charlson index were not significant.

In obesity class II, the association between RQ and REE/IBW was no longer significant (*p* = 0.97). Instead, VO_2_ emerged as a positive predictor (β = 0.107; *p* = 0.015), and SOFA score showed a positive association (β = 0.63; *p* = 0.002), suggesting that in moderate obesity the metabolic signal may shift toward oxidative demand and organ dysfunction burden. Male sex remained negatively associated with adjusted REE (β = −8.08; *p* < 0.001).

In obesity class III, none of the examined predictors reached statistical significance, although APACHE II showed a borderline inverse association (β = −0.43; *p* = 0.08). Given the small sample size (*n* = 16), these results should be interpreted cautiously, as the lack of significant predictors likely reflects limited statistical power and imprecise estimates rather than the absence of physiological determinants.

Structural heterogeneity across obesity grades was confirmed by the Chow test (F = 4.545, *p* = 0.0001), indicating that determinants of adjusted REE differ across adiposity strata.

### 3.6. Interactions Between Adiposity and Comorbidity Burden on Energy Metabolism

The interaction analysis between body mass index (BMI) and comorbidity burden, modeled through the BMI × Charlson term, revealed no statistically significant moderation effect of adjusted resting energy expenditure (REE/IBW). Neither the BMI × Charlson (β = −0.008; 95% CI: −0.075 to 0.059; *p* = 0.81) nor the BMI × RQ (β = −0.12; 95% CI: −1.28 to 1.03; *p* = 0.83) interaction terms reached significance, indicating that the influence of adiposity on metabolic rate is not materially altered by comorbidity burden or substrate oxidation efficiency.

Marginal effects analysis demonstrated that REE/IBW increased gradually with higher BMI across all Charlson strata, yet the magnitude of increase diminished with increasing comorbidity levels. Patients with no comorbidities (Charlson = 0) exhibited the steepest positive slope—from 27.6 kcal·kg^−1^·day^−1^ at BMI ≈ 19 to 36.7 kcal·kg^−1^·day^−1^ at BMI ≈ 53—whereas those with moderate (Charlson = 2) and high (Charlson = 5) comorbidity showed progressively flatter slopes (35.3 and 33.2 kcal·kg^−1^·day^−1^ at equivalent BMI values). Despite these differences in trajectory, overlapping 95% confidence intervals confirmed that comorbidity did not significantly modify the BMI–REE association.

From a mechanistic perspective, these findings support the notion of metabolic rigidity in critical illness: the capacity of energy metabolism to respond dynamically to variations in adiposity or disease burden appears blunted. The absence of interactive modulation implies that obesity-related metabolic attenuation is a structural, rather than compensatory, adaptation—reflecting a uniform suppression of oxidative and bioenergetic responses irrespective of comorbidity severity.

### 3.7. Nonlinear Association Between BMI and Adjusted Resting Energy Expenditure

**Figure 4:**
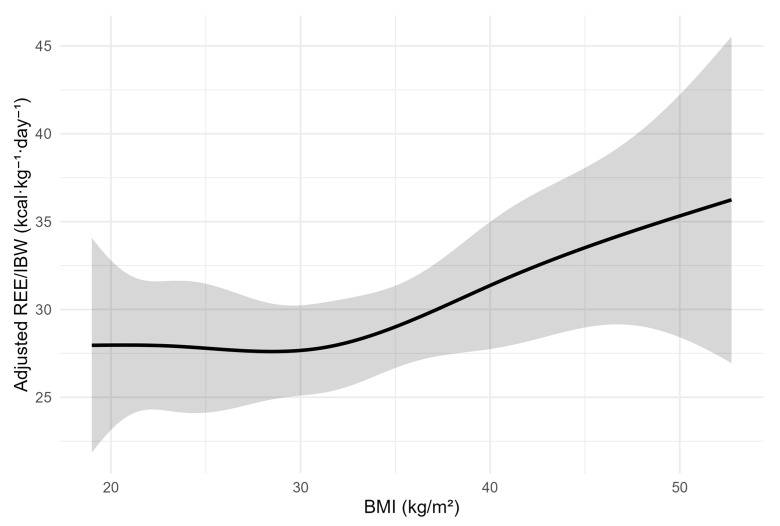
Nonlinear association between BMI and adjusted REE/IBW modeled with restricted cubic splines. The spline regression line (solid black) illustrates a modest nonlinear trend between BMI and REE/IBW, with a mild metabolic rebound at the upper end of the BMI spectrum. The shaded area represents the 95% confidence interval. The overall pattern supports adaptive thermometabolic stabilization at higher adiposity levels.

Restricted cubic spline modeling identified a modest nonlinear association between BMI and adjusted resting energy expenditure (REE/IBW). Only the upper curvature term reached statistical significance (β = 8.25; 95% CI 1.19–15.31; *p* = 0.022), suggesting a mild rebound in REE/IBW at the highest BMI values following a plateau across moderate adiposity ranges (Figure 4).

However, model comparison using the AICc criterion demonstrated that the linear specification provided the optimal balance between goodness of fit and parsimony (AICc = 681.0; ΔAICc = 0), whereas both the spline-based and interaction models showed substantially poorer fit (ΔAICc > 280). Accordingly, despite localized curvature at extreme BMI values, the linear model was retained as the primary analytic framework.

In this specification, age remained independently and inversely associated with REE/IBW (β = −0.108; 95% CI −0.196 to −0.021; *p* = 0.016), while sex, illness severity scores, and comorbidity burden were not significant covariates, although Charlson index approached borderline relevance (*p* = 0.051).

### 3.8. Sensitivity Analyses

Multiple sensitivity analyses were performed to assess the robustness of the primary REE/IBW model. Excluding measurements with non-physiological respiratory quotient values (<0.75 or >1.05) did not materially alter coefficient magnitude or direction, confirming the stability of the negative association between RQ and adjusted REE (β = −21.0; 95% CI −29.6 to −12.5; *p* < 0.001). Similarly, exclusion of patients with extreme BMI values (>60 kg/m^2^) yielded results consistent with the primary analysis.

Assessment of the incremental predictive value of comorbidity burden showed no improvement in model fit when the Charlson index was included as a continuous variable (ΔAICc = −1.82). When categorized into tertiles, moderate comorbidity was associated with a modest reduction in REE/IBW compared with low comorbidity (β = −2.95; 95% CI −5.88 to −0.02; *p* = 0.049).

Additional bioenergetic efficiency models demonstrated complementary patterns: REE/VO_2_ increased with higher RQ (β = 1.51; *p* < 0.001), whereas REE/VCO_2_ decreased as RQ increased (β = −5.39; *p* < 0.001), highlighting substrate-dependent asymmetry in oxidative metabolism. Multiple imputation analyses (MICE, m = 20) yielded estimates consistent with complete-case results, indicating minimal bias due to missing data.

### 3.9. Model Validation and Performance

Internal validation of the primary REE/IBW model demonstrated high predictive accuracy and stability. Ten-fold cross-validation yielded an RMSE of 2.88 kcal·kg^−1^·day^−1^, an MAE of 2.11 kcal·kg^−1^·day^−1^, a MAPE of 6.9%, and an R^2^ of 0.858, indicating that approximately 86% of the variability in adjusted REE was explained by the model.

Bootstrap resampling (1000 iterations) showed limited optimism, with an optimism-corrected R^2^ of 0.781 and RMSE of 3.73, supporting robustness against overfitting. Calibration analyses demonstrated excellent agreement between observed and predicted REE/IBW values across deciles, with a calibration slope of 1.00 (SE = 0.03; *p* < 0.001) and an intercept indistinguishable from zero, indicating minimal systematic bias.

Collectively, these validation procedures confirm that the proposed multivariable framework provides accurate, stable, and well-calibrated estimates of resting energy expenditure in critically ill patients with obesity.

## 4. Discussion

This study demonstrates that the choice of body weight–based normalization markedly influences the interpretation of REE in critically ill patients with obesity. While absolute REE remained relatively stable across BMI categories, REE normalized per kilogram of actual body weight (REE/kg) declined progressively with increasing adiposity, whereas indices adjusted by ideal or adjusted body weight (REE/IBW and REE/AdjBW) showed substantially less variability across BMI strata. These findings underscore that apparent metabolic differences across obesity phenotypes depend strongly on the denominator used, with important implications for clinical interpretation and nutritional prescription.

The progressive decline in REE/kg with increasing BMI reflects a pattern of relative hypometabolism, consistent with the lower metabolic activity of adipose tissue compared with fat-free mass [19,22]. This pattern, confirmed in multiple critically ill cohorts [23,24,25], may represent an adaptive energy-conserving mechanism that evolved to maintain metabolic homeostasis under stress [26,27]. However, such adaptation requires careful interpretation. Unlike the protective “obesity paradox” described for short-term mortality in certain critical illnesses [28,29,30], the metabolic inflexibility observed in this study likely reflects a maladaptive response rather than physiological protection. Supporting this view, recent evidence suggests that the obesity paradox is age-dependent and not uniformly applicable across critically ill populations [6,31]. In particular, patients with obesity class III in the present cohort exhibited marked metabolic rigidity, characterized by the absence of significant determinants of REE, a pattern more consistent with dysfunction than adaptive protection.

The contrast with reports of persistent hypermetabolism in COVID-19 [32], where REE may reach up to 150% of predicted values several weeks after ICU admission, underscores the heterogeneity of metabolic responses according to the etiology of critical illness. This variability highlights the limitations of uniform metabolic assumptions and reinforces the importance of direct measurement through indirect calorimetry.

A central finding of this study is the consistent inverse association between respiratory quotient (RQ) and adjusted REE across all normalization strategies. This association reflects differences in substrate utilization rather than a direct causal effect on energy expenditure. RQ, defined as the ratio of carbon dioxide production to oxygen consumption (VCO_2_/VO_2_), provides insight into substrate oxidation, with higher values indicating preferential carbohydrate oxidation and lower values reflecting lipid oxidation [24,31,33]. In the context of obesity, an elevated RQ despite sufficient or excessive energy stores is indicative of metabolic inflexibility, defined as an impaired ability to switch between lipid and carbohydrate oxidation in response to anelli metabolic demands [5,34].

The physiological basis of metabolic inflexibility is multifactorial. Obesity-associated insulin resistance disrupts the suppression of lipolysis and impairs cellular glucose uptake, leading to simultaneous substrate oversupply and inefficient utilization [34,35,36,37,38,39]. In skeletal muscle—the primary site of energy expenditure—ectopic lipid accumulation promotes lipotoxicity through bioactive lipid intermediates such as diacylglycerol and ceramides, which activate serine kinases, impair insulin receptor signaling, and ultimately reduce metabolic flexibility [34,40]. This mechanism underlies the well-described “athlete’s paradox,” wherein intramyocellular lipid accumulation is associated with metabolic dysfunction in insulin-resistant individuals but reflects adaptive metabolic capacity in trained athletes [34].

During critical illness, these chronic metabolic disturbances are further exacerbated by acute inflammatory stress. The observation that higher RQ values are associated with lower adjusted REE suggests that obligatory carbohydrate oxidation—a hallmark of metabolic inflexibility—is linked to reduced metabolic efficiency and constrained substrate utilization. This finding has direct clinical implications, as patients with elevated RQ values during indirect calorimetry may benefit from tailored macronutrient strategies, particularly reduced carbohydrate provision, to mitigate excessive glucose oxidation, lipogenesis, and their associated complications [41,42].

Stratified analyses revealed a progressive transition from preserved metabolic regulation to apparent rigidity across obesity grades. In obesity class I, metabolic control appeared relatively intact, with RQ and VCO_2_ emerging as key determinants of adjusted REE. In obesity class II, VO_2_ and SOFA score became relevant predictors, suggesting that oxidative demand and organ dysfunction increasingly influence energy metabolism. In obesity class III, the absence of significant predictors points toward pronounced metabolic rigidity or adaptive exhaustion. This pattern likely reflects convergent mechanisms, including adipocyte hypertrophy and inflammation [26], mitochondrial dysfunction limiting oxidative capacity, and loss of normal metabolic feedback. The near-significant inverse association with APACHE II in this group raises the possibility of a “ceiling effect,” whereby severe obesity constrains metabolic responsiveness regardless of acute physiological stress [43].

The association between male sex and lower adjusted REE after normalization aligns with previous findings by Drolz et al. [44], and may reflect fundamental sex-based differences in substrate utilization. Supporting this interpretation, a meta-analysis by Cano et al. demonstrated lower respiratory exchange ratios and greater fat oxidation in women [45], potentially driven by higher expression of genes involved in free fatty acid transport. These differences may translate into distinct relationships between gas exchange and calculated energy expenditure during critical illness.

Our findings add to the growing evidence highlighting the inadequacy of predictive equations in critically ill patients with obesity. Standard equations such as Harris–Benedict, Mifflin–St Jeor, and Penn State achieve acceptable accuracy in only 20–50% of cases when compared with measured REE [8,46]. A systematic review by Lambell et al. confirmed that ASPEN and ESPEN recommendations frequently under- or overestimate energy needs, with mean biases ranging from −48 to +150 kcal/day [47]. Importantly, equation performance deteriorates with increasing BMI severity. Mogensen et al. reported that among patients with morbid obesity, only 23% had predicted REE values within ±10% of measured expenditure, with substantial under- and overestimation [48]. The present study provides mechanistic insight into these limitations by demonstrating that determinants of REE differ fundamentally across obesity classes, precluding accurate prediction using population-derived formulas.

This investigation has several methodological strengths that boost confidence in its findings. The bicentric design, which involved recruiting two tertiary ICUs, improves population diversity and generalizability compared to single-center studies. The rigorous indirect calorimetry protocol, which included confirmation of hemodynamic and respiratory stability, achievement of steady state (with less than 10% variability in VO_2_ and VCO_2_), and physiological validation of respiratory quotient values (0.67–1.3), ensured high-quality metabolic measurements consistent with international standards [24,46,49].

Among limitations, the cross-sectional design precludes causal inference and temporal characterization of metabolic trajectories. Each patient was evaluated at a single time point, providing a metabolic “snapshot” that may not reflect dynamic changes over the course of the disease. Evidence shows that REE varies substantially: early sepsis typically exhibits hypermetabolism that resolves within 5–7 days [47], COVID-19 shows persistent hypermetabolism for weeks [32], and the transition to chronic critical illness is associated with normalization or hypometabolism [48]. Serial measurements over 7–14 days would provide critical insight into metabolic trajectories. Additionally, although bicentric, the study could not model center-level effects due to a lack of identifiers, preventing adjustment for institutional clustering; variability in clinical practices between centers may have introduced unmeasured confounding. The interpretation of sex-related differences in adjusted REE should be considered in light of normalization strategies and model specification, as associations reflect relative energy expenditure after adjustment rather than absolute metabolic demand.

Finally, the small sample size of patients with obesity class III (*n* = 16) limited statistical power for multivariable analyses in this subgroup. Consequently, the absence of significant predictors should be interpreted as exploratory rather than definitive. Larger, longitudinal studies with serial indirect calorimetry are needed to characterize metabolic trajectories across obesity phenotypes and to determine whether phenotype-guided nutritional strategies improve patient-centered outcomes. Randomized trials comparing indirect calorimetry–guided nutrition with predictive equation–based approaches are also warranted.

## 5. Conclusions

This study provides an integrative analysis of metabolic regulation in critically ill patients with obesity, demonstrating that resting energy expenditure (REE) is primarily determined by substrate oxidation efficiency rather than by anthropometric or clinical severity parameters. In all models, the respiratory quotient consistently emerged as a negative determinant of REE, reflecting impaired oxidative flexibility and decreased energetic efficiency in the context of critical illness.

The normalization of REE by different weight indices revealed that while absolute energy expenditure remains relatively constant across BMI categories, relative expenditure per kilogram decreases with increasing adiposity. This supports the concept of an adaptive hypometabolic phenotype in which excessive adipose mass contributes to a physiologically constrained energetic response. Notably, the linear specification of the BMI–REE relationship provided the best model fit, underscoring a proportional but limited influence of body composition on metabolic rate.

Comorbidity burden and clinical severity scores (SOFA, APACHE II) had minimal impact on energy metabolism after accounting for substrate oxidation variables. The absence of significant BMI × RQ and BMI × Charlson interactions further suggests that the suppression of metabolic flexibility in obesity is a structural adaptation rather than a dynamic response to comorbid disease or acute physiological stress.

Overall, these findings reinforce the necessity of individualized metabolic assessment in critical care nutrition. Reliance on predictive equations or weight-based adjustments may overlook the intrinsic metabolic heterogeneity driven by substrate utilization efficiency. Indirect calorimetry remains the most accurate approach to guide energy prescription in critically ill obese patients, ensuring both metabolic precision and clinical safety.

## Figures and Tables

**Table 1:** Bivariate comparison of metabolic and clinical parameters across BMI categories.

Characteristic	Normal (*n* = 29)	Overweight (*n* = 34)	Obesity I (*n* = 44)	Obesity II (*n* = 25)	Obesity III (*n* = 16)	Overall (*n* = 148)	*p*-Value
**REE (kcal/day)**	1871.4 ± 564.0	1822.6 ± 474.8	1848.8 ± 484.4	1866.4 ± 425.5	2071.9 ± 649.2	1874.3 ± 507.4	0.679
**REE/kg (kcal/kg·day)**	27.8 (22.9–35.0) ^a^	22.0 (18.8–28.5) ^ab^	20.0 (17.3–23.7) ^b^	17.8 (16.1–20.2) ^c^	16.9 (14.0–19.0) ^c^	20.5 (17.1–25.7)	<0.001
**REE/IBW (kcal/kg ideal body weight)**	29.2 ± 8.2	28.1 ± 6.6	29.6 ± 6.4	31.0 ± 5.8	34.5 ± 10.3	30.0 ± 7.4	0.139
**REE/AdjBW (kcal/kg adjusted body weight)**	29.1 ± 8.1	26.6 ± 6.2	26.7 ± 5.7	26.6 ± 5.0	27.1 ± 7.8	27.2 ± 6.5	0.712
**BMI (kg/m^2^)**	22.8 ± 1.7	27.4 ± 1.4	32.2 ± 1.3	37.5 ± 1.4	46.6 ± 4.5	31.7 ± 7.3	<0.001
**Age (years)**	69.0 (54.0–78.0)	67.0 (57.0–75.0)	65.5 (47.5–70.5)	66.0 (55.0–69.0)	68.0 (60.0–71.0)	66.0 (54.0–74.0)	0.540
**VO_2_ (mL/min)**	259.4 ± 82.0	250.1 ± 68.0	270.2 ± 77.8	263.7 ± 57.7	303.8 ± 98.7	265.9 ± 76.5	0.405
**VCO_2_ (mL/min)**	249.9 (194.0–307.6)	234.0 (194.6–311.7)	209.1 (175.0–261.0)	209.0 (189.0–257.0)	239.5 (194.7–307.3)	218.8 (189.0–294.6)	0.298
**Respiratory Quotient (RQ)**	1.0 ± 0.3 ^a^	1.0 ± 0.1 ^ab^	0.9 ± 0.1 ^c^	0.9 ± 0.1 ^ac^	0.9 ± 0.1 ^ac^	0.9 ± 0.2	<0.001
**SOFA score**	6.8 ± 3.9 ^ab^	5.6 ± 3.3 ^ab^	4.6 ± 4.0 ^a^	4.4 ± 2.9 ^a^	8.3 ± 3.2 ^b^	5.7 ± 3.8	0.002
**APACHE II score**	15.1 ± 5.6	14.7 ± 7.4	15.8 ± 9.3	15.3 ± 9.0	18.7 ± 6.9	15.6 ± 7.9	0.517
**Charlson Comorbidity Index**	2.0 (0.0–4.0)	2.0 (1.0–4.0)	3.0 (1.0–5.0)	4.0 (2.0–7.0)	4.0 (1.5–5.5)	3.0 (1.0–5.0)	0.192
**Sex, *n* (%)**							0.121
Female	11 (38%)	11 (32%)	18 (41%)	15 (60%)	10 (63%)	65 (44%)	
Male	18 (62%)	23 (68%)	26 (59%)	10 (40%)	6 (38%)	83 (56%)	

Values are presented as mean ± standard deviation (SD) or median (interquartile range, IQR), as appropriate. Overall *p*-values were obtained using one-way analysis of variance (ANOVA) for normally distributed variables and the Kruskal–Wallis test for non-normally distributed variables. When the overall test was statistically significant, pairwise comparisons were performed using Dunn’s test with Bonferroni correction. Groups sharing the same superscript letter do not differ significantly after Dunn–Bonferroni adjustment. Categorical variables were compared using the χ^2^ test.

## Data Availability

The datasets generated and analyzed during the current study are not publicly available due to institutional restrictions but are available from the corresponding author upon reasonable request.
